# Supplementary material for: An open-source framework for large-scale, flexible evaluation of biomedical text mining systems
Source: J Biomed Discov Collab. 2008 Jan 29;3:1. doi: 10.1186/1747-5333-3-1 (PMC2276192; doi:10.1186/1747-5333-3-1)
Supplement: Additional file 1 — Evaluation platform source code. The additional file contains the software evaluation framework discussed in this paper. The most current release can be downloaded from SourceForge.net [1]. See the accompanying README file for instructions on installation and use. Also included in the distribution is a collection of UIMA wrappers for some commonly used BioNLP tools and annotated corpora. [file 1747-5333-3-1-S1.zip › bionlp_uima_repository-1.0.1/docs/api/edu/uchsc/ccp/uima/annotation/CCPTextAnnotation.html]

CCPTextAnnotation (Center for Computational Pharmacology Util/UIMA API)


|  |  |  |
| --- | --- | --- |
| **Overview** \| **Package** \| **Class** \| **Tree** \| **Deprecated** \| **Index** \| **Help** | |  |
| **PREV CLASS**   **NEXT CLASS** | **FRAMES**    **NO FRAMES**     **All Classes** |  |
| SUMMARY: NESTED \| FIELD \| CONSTR \| METHOD | DETAIL: FIELD \| CONSTR \| METHOD |  |


---


## edu.uchsc.ccp.uima.annotation Class CCPTextAnnotation

```
java.lang.Object
  org.apache.uima.cas.impl.FeatureStructureImpl
      org.apache.uima.jcas.cas.TOP
          org.apache.uima.jcas.cas.AnnotationBase
              org.apache.uima.jcas.tcas.Annotation
                  edu.uchsc.ccp.uima.annotation.CCPAnnotation
                      edu.uchsc.ccp.uima.annotation.CCPTextAnnotation
```

**All Implemented Interfaces:**: Cloneable, org.apache.uima.cas.AnnotationBaseFS, org.apache.uima.cas.FeatureStructure, org.apache.uima.cas.text.AnnotationFS

**Direct Known Subclasses:**: CCPSemanticAnnotation, CCPSyntacticAnnotation

---

``` public class CCPTextAnnotation extends CCPAnnotation ```

The CCP TextAnnotation extends the base annotation class to include an annotation ID, the capability for multiple annotation spans, a link to the annotator responsible for generating the annotation, membership to annotation sets, and a link to a class mention which defines the class of this annotation.
Updated by JCasGen Tue Aug 28 16:44:10 MDT 2007
XML source: /home/williamb/eclipse/RELEASED\_SOURCE\_CODE/descriptors/typeSystem/CCPTypeSystem.xml

---

| **Field Summary** | |
| --- | --- |
| `static int` | `type` |
| `static int` | `typeIndexID` |

**Fields inherited from class org.apache.uima.jcas.cas.TOP**

`addr, jcasType`


| **Constructor Summary** | |
| --- | --- |
| `protected` | `CCPTextAnnotation()`             Never called. |
|  | `CCPTextAnnotation(int addr, org.apache.uima.jcas.cas.TOP_Type type)`             Internal - constructor used by generator |
|  | `CCPTextAnnotation(org.apache.uima.jcas.JCas jcas)` |
|  | `CCPTextAnnotation(org.apache.uima.jcas.JCas jcas, int begin, int end)` |


| **Method Summary** | |
| --- | --- |
| `int` | `getAnnotationID()`             getter for annotationID - gets The annotation ID provides a means for identifying a particular annotation. |
| `org.apache.uima.jcas.cas.FSArray` | `getAnnotationSets()`             getter for annotationSets - gets Annotation Sets provide an arbitrary means of categorizing and clustering annotations into groups. |
| `CCPAnnotationSet` | `getAnnotationSets(int i)`             indexed getter for annotationSets - gets an indexed value - Annotation Sets provide an arbitrary means of categorizing and clustering annotations into groups. |
| `CCPAnnotator` | `getAnnotator()`             getter for annotator - gets The annotator was responsible for generating this annotation. |
| `CCPClassMention` | `getClassMention()`             getter for classMention - gets The CCP ClassMention indicates the type (or class) for this annotation. |
| `int` | `getDocumentSectionID()`             getter for documentSectionID - gets The document section ID is optionally used to log what section of a document this annotation is from. |
| `int` | `getNumberOfSpans()`             getter for numberOfSpans - gets The number of spans comprising this annotation. |
| `org.apache.uima.jcas.cas.FSArray` | `getSpans()`             getter for spans - gets This FSArray stores the CCPSpans which comprise this annotation. |
| `CCPSpan` | `getSpans(int i)`             indexed getter for spans - gets an indexed value - This FSArray stores the CCPSpans which comprise this annotation. |
| `int` | `getTypeIndexID()` |
| `void` | `setAnnotationID(int v)`             setter for annotationID - sets The annotation ID provides a means for identifying a particular annotation. |
| `void` | `setAnnotationSets(org.apache.uima.jcas.cas.FSArray v)`             setter for annotationSets - sets Annotation Sets provide an arbitrary means of categorizing and clustering annotations into groups. |
| `void` | `setAnnotationSets(int i, CCPAnnotationSet v)`             indexed setter for annotationSets - sets an indexed value - Annotation Sets provide an arbitrary means of categorizing and clustering annotations into groups. |
| `void` | `setAnnotator(CCPAnnotator v)`             setter for annotator - sets The annotator was responsible for generating this annotation. |
| `void` | `setClassMention(CCPClassMention v)`             setter for classMention - sets The CCP ClassMention indicates the type (or class) for this annotation. |
| `void` | `setDocumentSectionID(int v)`             setter for documentSectionID - sets The document section ID is optionally used to log what section of a document this annotation is from. |
| `void` | `setNumberOfSpans(int v)`             setter for numberOfSpans - sets The number of spans comprising this annotation. |
| `void` | `setSpans(org.apache.uima.jcas.cas.FSArray v)`             setter for spans - sets This FSArray stores the CCPSpans which comprise this annotation. |
| `void` | `setSpans(int i, CCPSpan v)`             indexed setter for spans - sets an indexed value - This FSArray stores the CCPSpans which comprise this annotation. |

**Methods inherited from class org.apache.uima.jcas.tcas.Annotation**

`getBegin, getCoveredText, getEnd, getStart, setBegin, setEnd`

**Methods inherited from class org.apache.uima.jcas.cas.AnnotationBase**

`getSofa, getView`

**Methods inherited from class org.apache.uima.jcas.cas.TOP**

`addToIndexes, addToIndexes, equals, getAddress, getCAS, getCASImpl, getLowLevelCas, hashCode, removeFromIndexes, removeFromIndexes`

**Methods inherited from class org.apache.uima.cas.impl.FeatureStructureImpl**

`clone, getBooleanValue, getByteValue, getDoubleValue, getFeatureValue, getFeatureValueAsString, getFloatValue, getIntValue, getLongValue, getShortValue, getStringValue, getType, prettyPrint, prettyPrint, prettyPrint, setBooleanValue, setByteValue, setDoubleValue, setFeatureValue, setFeatureValueFromString, setFloatValue, setIntValue, setLongValue, setShortValue, setStringValue, toString, toString`

**Methods inherited from class java.lang.Object**

`finalize, getClass, notify, notifyAll, wait, wait, wait`

**Methods inherited from interface org.apache.uima.cas.AnnotationBaseFS**

`getView`

**Methods inherited from interface org.apache.uima.cas.FeatureStructure**

`clone, equals, getBooleanValue, getByteValue, getCAS, getDoubleValue, getFeatureValue, getFeatureValueAsString, getFloatValue, getIntValue, getLongValue, getShortValue, getStringValue, getType, hashCode, setBooleanValue, setByteValue, setDoubleValue, setFeatureValue, setFeatureValueFromString, setFloatValue, setIntValue, setLongValue, setShortValue, setStringValue`

**Field Detail**

### typeIndexID

```
public static final int typeIndexID
```

---


### type

```
public static final int type
```


**Constructor Detail**

### CCPTextAnnotation

```
protected CCPTextAnnotation()
```

:   Never called. Disable default constructor

---


### CCPTextAnnotation

```
public CCPTextAnnotation(int addr,
                         org.apache.uima.jcas.cas.TOP_Type type)
```

:   Internal - constructor used by generator

---


### CCPTextAnnotation

```
public CCPTextAnnotation(org.apache.uima.jcas.JCas jcas)
```

---


### CCPTextAnnotation

```
public CCPTextAnnotation(org.apache.uima.jcas.JCas jcas,
                         int begin,
                         int end)
```


**Method Detail**

### getTypeIndexID

```
public int getTypeIndexID()
```

:   **Overrides:**: `getTypeIndexID` in class `CCPAnnotation`

---


### getAnnotationID

```
public int getAnnotationID()
```

:   getter for annotationID - gets The annotation ID provides a means for identifying a particular annotation. Setting this ID is optional. The default value should be -1.

---


### setAnnotationID

```
public void setAnnotationID(int v)
```

:   setter for annotationID - sets The annotation ID provides a means for identifying a particular annotation. Setting this ID is optional. The default value should be -1.

---


### getAnnotator

```
public CCPAnnotator getAnnotator()
```

:   getter for annotator - gets The annotator was responsible for generating this annotation.

---


### setAnnotator

```
public void setAnnotator(CCPAnnotator v)
```

:   setter for annotator - sets The annotator was responsible for generating this annotation.

---


### getDocumentSectionID

```
public int getDocumentSectionID()
```

:   getter for documentSectionID - gets The document section ID is optionally used to log what section of a document this annotation is from. Values can be specified by the user. See edu.uchsc.ccp.util.nlp.document.DocumentSectionTypes for a few common sections.

---


### setDocumentSectionID

```
public void setDocumentSectionID(int v)
```

:   setter for documentSectionID - sets The document section ID is optionally used to log what section of a document this annotation is from. Values can be specified by the user. See edu.uchsc.ccp.util.nlp.document.DocumentSectionTypes for a few common sections.

---


### getAnnotationSets

```
public org.apache.uima.jcas.cas.FSArray getAnnotationSets()
```

:   getter for annotationSets - gets Annotation Sets provide an arbitrary means of categorizing and clustering annotations into groups.

---


### setAnnotationSets

```
public void setAnnotationSets(org.apache.uima.jcas.cas.FSArray v)
```

:   setter for annotationSets - sets Annotation Sets provide an arbitrary means of categorizing and clustering annotations into groups.

---


### getAnnotationSets

```
public CCPAnnotationSet getAnnotationSets(int i)
```

:   indexed getter for annotationSets - gets an indexed value - Annotation Sets provide an arbitrary means of categorizing and clustering annotations into groups.

---


### setAnnotationSets

```
public void setAnnotationSets(int i,
                              CCPAnnotationSet v)
```

:   indexed setter for annotationSets - sets an indexed value - Annotation Sets provide an arbitrary means of categorizing and clustering annotations into groups.

---


### getNumberOfSpans

```
public int getNumberOfSpans()
```

:   getter for numberOfSpans - gets The number of spans comprising this annotation. The CCP TextAnnotation allows the use of multiple spans for a single annotation.

---


### setNumberOfSpans

```
public void setNumberOfSpans(int v)
```

:   setter for numberOfSpans - sets The number of spans comprising this annotation. The CCP TextAnnotation allows the use of multiple spans for a single annotation.

---


### getSpans

```
public org.apache.uima.jcas.cas.FSArray getSpans()
```

:   getter for spans - gets This FSArray stores the CCPSpans which comprise this annotation. It should be noted that for an annotation with multiple spans, the default begin and end fields are set to the beginning of the first span and the end of the final span, respectively.

---


### setSpans

```
public void setSpans(org.apache.uima.jcas.cas.FSArray v)
```

:   setter for spans - sets This FSArray stores the CCPSpans which comprise this annotation. It should be noted that for an annotation with multiple spans, the default begin and end fields are set to the beginning of the first span and the end of the final span, respectively.

---


### getSpans

```
public CCPSpan getSpans(int i)
```

:   indexed getter for spans - gets an indexed value - This FSArray stores the CCPSpans which comprise this annotation. It should be noted that for an annotation with multiple spans, the default begin and end fields are set to the beginning of the first span and the end of the final span, respectively.

---


### setSpans

```
public void setSpans(int i,
                     CCPSpan v)
```

:   indexed setter for spans - sets an indexed value - This FSArray stores the CCPSpans which comprise this annotation. It should be noted that for an annotation with multiple spans, the default begin and end fields are set to the beginning of the first span and the end of the final span, respectively.

---


### getClassMention

```
public CCPClassMention getClassMention()
```

:   getter for classMention - gets The CCP ClassMention indicates the type (or class) for this annotation.

---


### setClassMention

```
public void setClassMention(CCPClassMention v)
```

:   setter for classMention - sets The CCP ClassMention indicates the type (or class) for this annotation.


---


|  |  |  |
| --- | --- | --- |
| **Overview** \| **Package** \| **Class** \| **Tree** \| **Deprecated** \| **Index** \| **Help** | |  |
| **PREV CLASS**   **NEXT CLASS** | **FRAMES**    **NO FRAMES**     **All Classes** |  |
| SUMMARY: NESTED \| FIELD \| CONSTR \| METHOD | DETAIL: FIELD \| CONSTR \| METHOD |  |


---
